# *In Vivo* Antitumoral Effects of Linseed Oil and Its Combination With Doxorubicin

**DOI:** 10.3389/fphar.2022.882197

**Published:** 2022-06-21

**Authors:** Oleg Shadyro, Anna Sosnovskaya, Irina Edimecheva, Lana Ihnatovich, Boris Dubovik, Sergei Krasny, Dmitry Tzerkovsky, Egor Protopovich

**Affiliations:** ^1^ Laboratory of Chemistry of Free-Radical Processes, Research Institute for Physical and Chemical Problems, Belarusian State University, Minsk, Belarus; ^2^ Department of Chemistry, Belarusian State University, Minsk, Belarus; ^3^ Department of Pharmacology, Belarusian State Medical University, Minsk, Belarus; ^4^ Laboratory of Photodynamic Therapy and Hyperthermia, N.N. Alexandrov National Cancer Center, Lesnoy, Belarus

**Keywords:** linseed oil, doxorubicin, Pliss lymphosarcoma, Lewis lung adenocarcinoma, combination therapy, antitumor effect, complete tumor regression

## Abstract

Linseed oil (LO) is known for its exceptional nutritional value due to the high content of alpha-linolenic acid (ALA), an essential omega-3 polyunsaturated fatty acid; its anticarcinogenic effect has been established in several experimental and epidemiological studies. As an adjuvant of chemotherapeutic agents, LO and other ALA-rich vegetable oils have been studied in only a handful of studies at the experimental level. However, the efficacy of antitumoral therapy using doxorubicin (Dox) in combination with ALA and ALA-rich substrates has not yet been investigated. In this work, the antitumor activity of LO in a wide dose range was studied with monotherapy and combined with Dox in animal models with Pliss lymphosarcoma (PLS) and Lewis lung adenocarcinoma (LLC). It was founded the daily oral administration of LO (1, 3, and 10 ml per 1 kg) to rats (PLS) and 6 ml/kg to mice (LLC) for 11–12 days from 7 days after subcutaneous transplantation of tumors has a stable statistically significant effect on the dynamics of tumor growth, reducing the intensity of tumor growth and increasing the frequency of complete tumor regressions (CR) compared with the control. LO showed high antimetastatic activity in the LLC model. Furthermore, LO at a dose of 3 ml/kg potentiates the antitumor effect of Dox in the PLS model, reducing the volume of tumors at the end of treatment by 2.0 times (*p* = 0.013), the value of the tumor growth index by 1.6 times (*p* < 0.03) and increasing the frequency of CR 60 days after the start of therapy by 3.5 times (*p* = 0.019) compared with the use of Dox alone. The combination of Dox and LO or fish oil allows growing efficiency therapy of LLC in comparison with Dox alone, increasing the frequency of CR to 73.68% and 94.4%, respectively, and reducing the frequency of metastasis to zero.

## Introduction

Chemotherapy as well as pharmacology in general is based on a fundamental principle of stochastic interaction of chemical agents with “biotargets.” Based on this concept and taking into account the unique adaptive properties of cancerous cells [“hallmarks of cancer” (Fouad and Aanei, 2017)], the affinity constant of antitumor agents to cancer molecular targets should be very high. In terms of the log-kill hypothesis, it means that a log-kill rate should reach the value of 6–7 and higher to achieve the eradication of tumor elements in one or two therapeutic cycles before cancerous cells develop resistance to treatment. In reality, such levels of selectivity remain unachievable. Despite the appearance of targeted antitumor drugs, which significantly increase the effectiveness of treatment of several tumor diseases (Hanahan, 2014; Olsson, 2019; Sung et al., 2021), the process of developing new antitumor drugs becomes more expensive and less efficient. Overall, the area of antitumor therapy is still far from a cardinal breakthrough. In this regard, along with the improvement of combined chemotherapy, which is a standard practice in oncology, and the search for new ways in the treatment of tumors (Falzone et al., 2018), approaches aimed at improving the effectiveness of common antitumor agents are promising. The latter is based on the regulation of the tumor cell biology features that are important for the initial and acquired tolerance of tumors to chemotherapy. Remarkably, this strategy works not only at the experimental but also at the clinical level, when conventional drugs for various purposes (“drug repositioning”) (Yang et al., 2016; Falzone et al., 2018; Shah and Stonier, 2019; Dinić et al., 2020) and other available natural and synthetic compounds, e.g., bioflavonoids and nutraceuticals (Story, 2020; Liskova et al., 2021; Yuen and Tsao, 2021), complement popular antitumor agents.

Marine omega-3 polyunsaturated fatty acids (n-3 PUFAs), namely eicosapentaenoic (EPA) and docosahexaenoic acids (DHA) attract particular attention. These widely studied and safe nutrients, being the components of fish oil (FO), have a wide range of therapeutic and prophylactic properties, including antitumor activity. For instance, it has been shown that the consumption of n-3 PUFAs (as pure compounds or as a part of FO) inversely correlates with the risk of various cancerous tumors, including the most common prostate, breast, and colorectal cancer (Terry et al., 2001; Pauwels and Kairemo, 2008; Wu et al., 2012; Fabian et al., 2015; Lin et al., 2019; Aglago et al., 2020). In addition, the combined use of EPA and DHA with various antiblastoma agents enhances their effect in experiments and clinical conditions in several solid tumors and hemablastoses (Cockbain et al., 2012; Jing et al., 2013; Merendino et al., 2013; Lee et al., 2017; Zhang and Chen, 2020; Chen et al., 2021; Newell et al., 2021).

Although the nature of these effects is not fully disclosed, it is evident that n-3 PUFAs implement multiple antitumor mechanisms critical for the generation and progression of tumors. Such mechanisms may include induction of apoptosis, cell cycle arrest at the level of different signaling events, inhibition of tumor angiogenesis, metastasis, inflammatory reaction of the microenvironment, epigenetic and other malfunctions (D’Eliseo and Velotti, 2016; Newell et al., 2017; Ravacci et al., 2013; Calviello et al., 2007; Nabavi et al., 2015; Corsetto et al., 2017). PUFAs, including n-3 PUFAs, are highly susceptible to lipid (per)oxidation (LPO). So an increase in LPO and subsequent accumulation of secondary LPO products possessing cytostatic and cytotoxic properties may, at least partially, define the antitumor effect of n-3 PUFAs in conditions of relative oxygen deficiency (one of the “hallmarks of cancer”) (Fouad and Aanei, 2017). It can be assumed that in the presence of n-3 PUFAs, cytostatic oxidants such as doxorubicin (Dox) more effectively induce an increase in the intracellular concentration of reactive oxygen species (ROS), cytotoxic products of oxidation and oxidative destruction of biomolecules, and regulate other equally important free-radical processes in cancer cells. Such alternatives, arising from our studies of the patterns of free-radical transformations of bioorganic compounds in hypoxic conditions, will be considered further when discussing the results.

The most common nutrient in the n-3 PUFA family is alpha-linolenic acid (ALA) (С18: 3). ALA is an essential fatty acid necessary for the biosynthesis of several vital structural and signaling molecules, including long-chain, more unsaturated n-3 PUFAs like EPA (С20: 5) and DHA (С22: 6). EPA and DHA are biosynthesized by particular elongases and desaturases, which also competitively transform n-6 linoleic acid (С18: 2) into n-6 arachidonic acid (С20: 4) (Burdge et al., 2002; Burdge and Calder, 2006). But since linoleic acid in the diet usually contains 10–20 times more than ALA, the conversion of ALA into EPA and DHA in humans is estimated by different authors at the level of 1.5%–15% (Burdge and Calder, 2006; Domenichiello et al., 2015). Consequently, the introduction of ALA into the diet, which increases the ratio of n-3/n-6 PUFAs, can ensure the necessary effective level of long-chain n-3 PUFAs, more unsaturated than ALA, which is considered a significant factor in their antitumor activity (Nabavi et al., 2015).

ALA is found in substantial amounts in some seeds, nuts, and vegetable oils. ALA-rich plant sources are more accessible and plentiful and may serve as a suitable alternative to fish lipids. Linseed oil (LO) obtained from flax seeds is especially rich in ALA. In addition, the developed and implemented industrial technology of LO stabilization (Shadyro et al., 2017) ensures the long-term preservation of this source of n-3 PUFAs. At the same time, the effects of vegetable n-3 PUFAs in cancer have not been studied as widely at the epidemiological level as of their marine homologues, and the data in this area are contradictory (Brouwer et al., 2004; De Lorgeril and Salen, 2004; Simon et al., 2009; Harris et al., 2021; Naghshi et al., 2021). Animal model studies using LO and other ALA-rich vegetable oils confirm their anticancer and antimetastatic effects in prostate, breast, cervical, rectum cancer, Walker 256 carcinosarcoma, and melanoma (Truan et al., 2010; Han et al., 2015; Mason et al., 2015; Schiessel et al., 2015; Li et al., 2017; Vara-Messler et al., 2017; DeLuca et al., 2018; Deshpande et al., 2019). At the experimental level, ALA as an adjuvant of chemotherapeutic agents has been studied in only a handful of studies (Yu et al., 2013; Mason et al., 2015; Deshpande et al., 2019), and the question of whether ALA can replace marine n-3 PUFAs or it has independent effects remains open.

Based on the above premises, the purpose of this work was to study the antitumor activity of LO in a wide dose range in animal model experiments with LO administered as a single agent and combined with Dox.

## Materials and Methods

### Chemicals

Doxorubicin hydrochloride (Dox) was obtained from Belmedpreparaty (Minsk, Belarus). Linseed oil was purchased from Club Farm-Eco Company (Drogichin, Belarus). Fish oil was purchased from Lysi (Reykjavik, Iceland). Characteristics of LO and FO including fatty acid composition were determined as described in (Shadyro et al., 2017) and presented in Supplementary Table S1.

Hanks’ Balanced Salt solution (HBSS) was acquired from LT Biotech Ltd. (Vilnius, Lithuania). The saline (0.9% NaCl solution) was prepared in the laboratory.

### Animal Models

Mongrel rats (Wistar) and mice (C57BL/6) used for the study were of a body weight 140–170 g and 20–26 g, respectively, and between 2.5 and 3 months of age. The ratio of male and female rats was 7:3; the group of mice had comparable numbers of males and females. They were kept under standard conditions with food and water ad libitum. When animals showed terminal signs, they were euthanized in compliance with the AVMA Guidelines for the Euthanasia of Animals (Hubrecht and Carter, 2019; AVMA Guidelines for the Euthanasia of Animals, 2020; Directive 2010/63/EU on the protection of animals used for scientific purposes, 2010).

### Tumor Xenograft Models

Pliss lymphosarcoma (PLS) (Vasiliev et al., 2009) strain was obtained from the Russian Cell Culture Collection, Institute of Cytology of the Russian Academy of Sciences (St. Petersburg, Russia). Lewis lung carcinoma (LLC) was provided by the N.N. Blokhin Russian Cancer Research Center (Moscow, Russia).

PLS and LLC xenografts were grown in rats and mice, respectively. Briefly, PLS (0.5 ml, 10% suspension in HBSS) was injected subcutaneously in the left inguinal region in rats. LLC tumor cells (0.2–0.3 ml, 4 × 10^6^ cells/ml suspension in HBSS) were injected under the skin of the back of mice.

### Animal Treatment

Compound treatment was started on the 7th–18th (for PLS) or the 7th–19th (for LLC) day after the injection, when the tumor reached a minimum diameter of 3–4 mm. Prior to the treatment, the animals were randomized into experimental groups. Dox (5 mg/kg of animal body weight) was administered once at the start of treatment by i.p. injection. LO and FO (various doses), as well as vehicle control (saline, 10 ml/kg of body weight), were administered orally (once daily). The treatment and daily animal assessment were carried out in a blinded fashion.

The PLS model study was performed in three experiments. The 1st one was a pilot study aiming to select optimal doses for LO, where rats were divided into eight groups (6–7 animals each). For the latter, the number of animals per group was expanded to 10–11 individuals. Group I was control, group II was treated with Dox, groups III-V (LO 1, LO 3, LO 10) were receiving LO (1, 3, and 10 ml per 1 kg), and groups VI-VIII (Dox + LO 1, Dox+LO 3, Dox+LO 10) were subjected to the combined Dox (5 mg/kg) + LO (1, 3, and 10 ml per 1 kg) treatment. LO at a dose having the greatest potentiating effect on Dox antitumor activity was used for experiment 3. To confirm the identified effects was performed with four groups (10 animals each) using control (group I), Dox (group II), 3 ml/kg LO (group IV), and Dox + 3 ml/kg LO (group VII) treatment options.

The LCC study involved six groups of mice (10–11 animals each): group I was control, group II (LO) were receiving LO (6 ml/kg), group III was treated with Dox (5 mg/kg), group IV (Dox+LO) were subjected to the combined Dox (5 mg/kg) + LO (6 ml/kg), group V (FO) were receiving FO (6 ml/kg), group VI (Dox+FO) were subjected to the combined Dox (5 mg/kg) + FO (6 ml/kg). The experiment was conducted twice.

Within 2 weeks after the start of the experiment (administration of drugs), during which the linear dimensions of tumors were measured, euthanasia was carried out in the case of rapid progression (large tumor sizes, decay and infection of tumors), as well as with the appearance of pronounced cachexia. The remaining animals were under observation for 60 days to assess the frequency of complete regressions: they were both with and without tumors. If the above conditions appeared within 11–12 to 60 days after the start of the experiment, the animals were taken out of work. If there was a stabilization of tumor growth with a minimal increase in tumor volumes or with their reduction, animals were observed.

### Tumor Characterization Parameters

Antitumor activity of the treatment was assessed using the following parameters:

1) Dynamic changes in tumor volume over the whole course of treatment. Three mutually perpendicular tumor diameters (d_1_, d_2_, and d_3_) were measured every 2–3 days and tumor volume (V, cm^3^/mm^3^) was calculated according to the formula:

$$\text{V}=\frac{1}{6}{\text{πd}}_{1}\;\;\cdot \;{\text{d}}_{2}\cdot \;{\text{d}}_{3}$$ (1)

The size of the tumor was measured using a special caliper. The tumors were measured for 11–12 days from the start of the therapy, after which the mouse and rats remained under observation for 60 days.

2) Tumor growth inhibition coefficient (IC):

$$\text{IC}=\frac{\left({\text{V}}_{\text{C}}-{\text{V}}_{\text{0}}\right)}{{\text{V}}_{\text{C}}}100\%$$ (2)

where V_o_ and V_c_ are average tumor volumes (cm^3^/mm^3^) in experimental and control groups, respectively. IC > 50% was considered biologically significant.

3) Tumor growth index (TGI) as an integral indicator of antitumor activity, which takes into account not only the intensity of the effect, but also its stability (Stukov et al., 2001; Duan et al., 2012):

$$\text{TGI}=\frac{{\text{S}}_{\text{0}}}{{\text{S}}_{\text{C}}}\;100\%$$ (3)

where S_o_ and S_c_ are areas under the kinetic curve of tumor growth in experimental and control groups, respectively. S_o_ and S_c_ were calculated for the whole period of treatment (11–12 days) using a statistical package of OriginPro 2015 SR1 Build 9.2.257 (OriginPro Corporation, United States).

4) Frequency of complete tumor regression (CR) was calculated as % of the total number of animals per group. It was evaluated at the end of treatment and 60 days after the start of treatment if visual and palpation signs of tumor growth were absent.
5) Severity of the metastatic process (for LLC model), which was assessed using the following indicators: 1) frequency of tumor metastasis in % of animals with metastases to the total number of animals per group; 2) average number of metastases per animal in the group; 3) degree of lung metastasis (0: no metastases; 1: < 10 metastatic nodes with a diameter ≤ 1 mm; 2: 10–30 metastatic nodes; 3: ≥ 30 metastatic nodes of various sizes; 4: ≤ 100 metastatic nodes without drain growth; 5: ≥ 100 metastatic nodes and the presence of solid tumor nodes). The number of metastases was determined at the end of the treatment. The metastasis inhibition index (MI) was calculated by the formula:

$$\text{MI}=\frac{\left({\text{A}}_{\text{C}}\cdot {\text{B}}_{\text{C}}\right)-\left(\text{A}\cdot \text{B}\right)}{{\text{A}}_{\text{C}}\cdot {\text{B}}_{\text{C}}}100\%$$ (4)

where A and A_c_ are frequencies of lung metastasis in experimental and control groups, respectively; B and B_c_ are average numbers of lung metastases per animal in experimental and control groups, respectively.

Metastasis was determined by microscopic method. Animals were killed by chloroform vapors. The chest was being opened. The lungs were completely excised and washed with saline solution, after which they were placed in Carnoys liquid for 0.5–1 h for fixation. Later, using a binocular microscope, the number of metastases was estimated both on the surface of the tissue and in its depth in both lungs, and not their parts. The frequency of tumor metastasis was determined in % of laboratory animals with metastases relative to the total number of animals in the group.

### Statistical Analysis

Statistical analysis and data visualization were performed using Statistica 12.5 (StatSoft, United States). The experiment was repeated several times, the experimental data obtained from different series were combined into one dataset. All the combined data series were checked for uniformity. All batches to be combined are checked for homogeneity: the Shapiro–Wilk test (the check for the type of distribution), the Levene’s test (check for the equality of variances), the Student criterion (for equal variances), or the Mann-Whitney criterion (for unequal variances) for pairwise comparison of data from different series. The combined series had no statistically significant differences (*p* ≥ 0.05), and they were attributed to one sample population.

Tumor volume values for the PLS model (rats) had a Gauss distribution (*p* ≥ 0.05, Shapiro-Wilk’s test), equal variance (*p* ≥ 0.05, Levene’s test), and were subjected to the univariate analysis (one-way ANOVA) with a posteriori multiple Tukey comparisons.

Tumor volume values for the LLC model (mice) did not have a normal distribution; therefore, the Mann-Whitney nonparametric criterion was used to identify statistically significant effects. The obtained results are presented as median and upper and lower quartile values.

The relative risk (RR) and 95% confidence interval (CI) were calculated using the Cox regression model. Comparisons were made using the 2 × 2 contingency table from Statistica 12.5.

The differences between treatment options were considered statistically significant at *p* < 0.05.

### Ethics Approval

All animal experiments were approved by the Ethics Committee of the N.N. Alexandrov National Cancer Center of Belarus (the experimental protocol No. 163 from 22.05.2020) and carried out in compliance with the requirements of the European Convention about the maintenance, feeding, and care of experimental animals (European convention for the protection of vertebrate animals used for experimental and other scientific purposes, 1986).

## Results

### Linseed Oil Enhances the Antitumor Activity of Doxorubicin in the Treatment of Pliss Lymphosarcoma

Antitumor efficacy of various doses of LO (1, 3, 10 ml/kg), Dox (5 mg/kg), and their combinations were studied in the PLS model in rats. Taking into account the content of ALA (omega-3) in LO (Supplementary Table S1) and the oil density (0.93 g/cm^3^), the animals received daily (52.05 ± 2.70); (156.15 ± 8.15) and (520.50 ± 27.24) mg ALA/100 g body weight, respectively (the results were expressed as mean ± SD). All treatment options did not cause any statistically significant perturbations of animal body weight.

**FIGURE 1:**
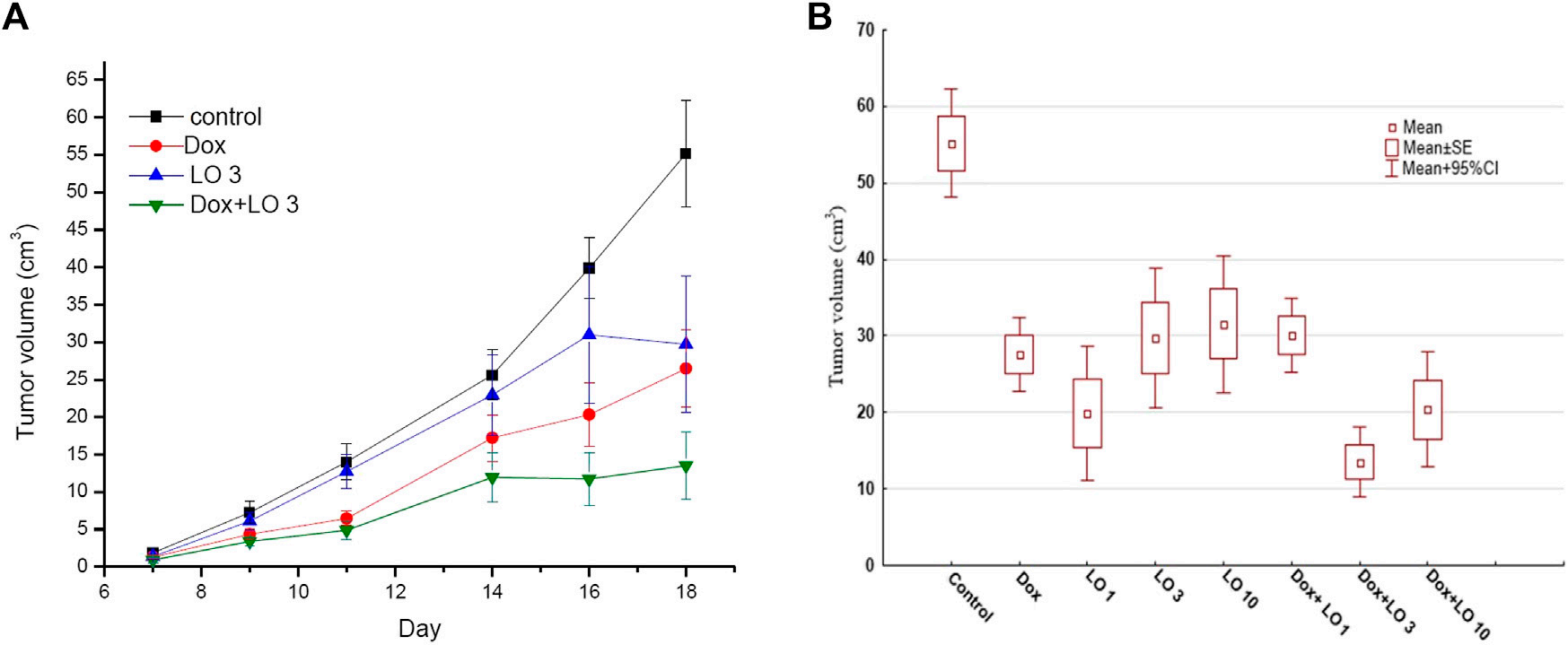
The dynamics changes of PLS tumor volume over the whole course of treatment **(A)**; the tumor volume 11 days after the start of treatment **(B)**. The data obtained three independent experiments for Control, Dox, LO 3, and Dox+LO 3 groups. The values represent the mean ± 95% CI **(A,B)**.

Figure 1 shows the dynamics of tumor growth in several experimental groups (Figure 1A) and the values of PLS tumor volumes 11 days from the start of therapy for the studied groups of animals (Figure 1B).

According to Figure 1, LO with daily oral administration at a dose of 3 ml/kg demonstrated marked antitumor activity, inhibiting the growth of PLS. The most effective is the combined use of cytostatic Dox and LO. Table 1 shows descriptive statistics for all the studied groups at the end of treatment (11 days after the start of treatment).

**TABLE 1:** Descriptive statistics for the studied groups of animals according to values on the volume of PLS tumors after 11 days from the start of treatment.

Variable	Valid N	Mean	−95% CI	+95% CI	Trimmed mean (±5%)	Std. Dev.	SE	Median
Control	21	55.18	47.65	62.71	54.60	16.54	3.61	52.56
Dox	23	27.59	22.44	32.74	27.55	11.92	2.48	28.68
LО 1	14	19.93	10.30	29.56	19.08	16.68	4.46	24.05
LО 3	24	29.71	20.10	39.33	28.98	22.76	4.65	32.06
LО 10	13	31.56	21.64	41.48	32.58	16.42	4.55	32.64
Dox+LO 1	14	30.11	24.72	35.49	29.84	9.32	2.49	30.61
Dox+LO 3	25	13.51	8.79	18.24	13.26	11.45	2.29	16.39
Dox+LO 10	15	20.39	12.17	28.60	20.06	14.84	3.83	20.82

N‒the number of animals in groups at the end of treatment. Died and euthanized animals are not included.

Since the data on tumor volumes in each group for the whole period of treatment had a normal distribution, the Tukey test was chosen for group comparison. Of note, tests used for pairwise comparisons with normal distribution, such as Newman-Keuls, LSD, Duncan tests, showed similar results. The levels of statistical significance of pairwise comparison (Tukey’s test) of all eight groups at the end of treatment are presented in Supplementary Table S2. According to Tukey’s test, all seven treatment options resulted in statistically significant (*p* ≤ 0.001) tumor growth inhibition compared to the control. A statistically significant difference in the antitumor effect of the study doses of LO and its combinations with Dox was observed from the 9th day of treatment. LO at doses of 1–10 ml/kg caused a decrease in tumor volume by 1.7–2.8 times compared to the control at the end of treatment. According to the antitumor effect, the studied LO doses statistically did not differ significantly from each other and the Dox used alone (Supplementary Table S2). When compared with the group treated with Dox alone, statistically significant differences in tumor volume values occurred only for the control and Dox+LO 3 groups. By the end of therapy, the combination of Dox and LO (3 ml/kg) reduced the volume of the PLS tumor by 4.1 times compared with the control (*p* < 0.001) and by 2.0 times compared with the Dox used alone (*p* = 0.013). Other doses of LO (1 and 10 ml/kg) in a combined treatment with Dox had a statistically significant (*p* < 0.001) antitumor effect compared with the control but did not give markedly different results compared with the use of Dox alone (Supplementary Table S2).

**FIGURE 2:**
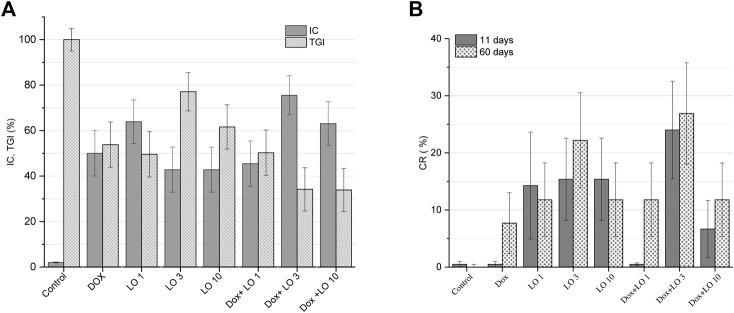
PLS tumor growth inhibition coefficients (IC) 11 days after the start of treatment and tumor growth indices (TGI) corresponding to the whole treatment period **(A)**. Complete tumor regression (CR) frequencies calculated 11 and 60 days after the start of treatment **(B)**. The values represent the mean % ± SE **(A,B)**. The data correspond to two and three independent experiments.

Analysis of IC values for different treatment options revealed that only Dox + LO 3 and Dox + LO 10 exceeded the threshold of biological significance (IC > 50%) in Figure 2A. IC for the combined use of Dox+LO 3 at the end of treatment was the highest and equal to (75.51 ± 8.60)%. It differed significantly (*p* = 0.028, Student’s *t*-test) from IC of Dox equal to (50.00 ± 10.00)%, which did not surpassed the threshold value.

The therapeutic effect of LO was also evaluated on the basis of the TGI, which reflects the entire tumor growth curve in one number and allows for easier comparison between experimental groups (Stukov et al., 2001; Duan et al., 2012). Based on TGI values, only the combined use of Dox and LO (3 and 10 ml/kg) showed statistically significant (*p* < 0.021, Student’s *t*-test) inhibition of tumor growth by 1.60 times compared to Dox administered alone (Figure 2A). CR frequencies of PLS tumors registered after 11 and 60 days from the start of treatment are shown in Figure 2B. Among all studied treatment options, the combined use of Dox + LO 3 demonstrated the highest CR value equal to (24,00 ± 7,22)% and (26.91 ± 7.50)% 11 and 60 days following the start of therapy, respectively, and for the post-treatment observation, it was 3.49 times higher (*p* = 0.019, Student’s *t*-test) than the same of Dox. Of note, the use of Dox as a single agent showed zero CR value 11 days after the start of treatment. Overall, statistically significant differences (*p* < 0.012, Student’s *t*-test) in CR values were obtained for LO 3, LO 10, and Dox+LO 3 11 days after the start of treatment compared with the control group. 60 days after the start of therapy, all experimental groups differed significantly (*p* ≤ 0.02, Student’s *t*-test) in CR values from the control group (Figure 2B).

**FIGURE 3:**
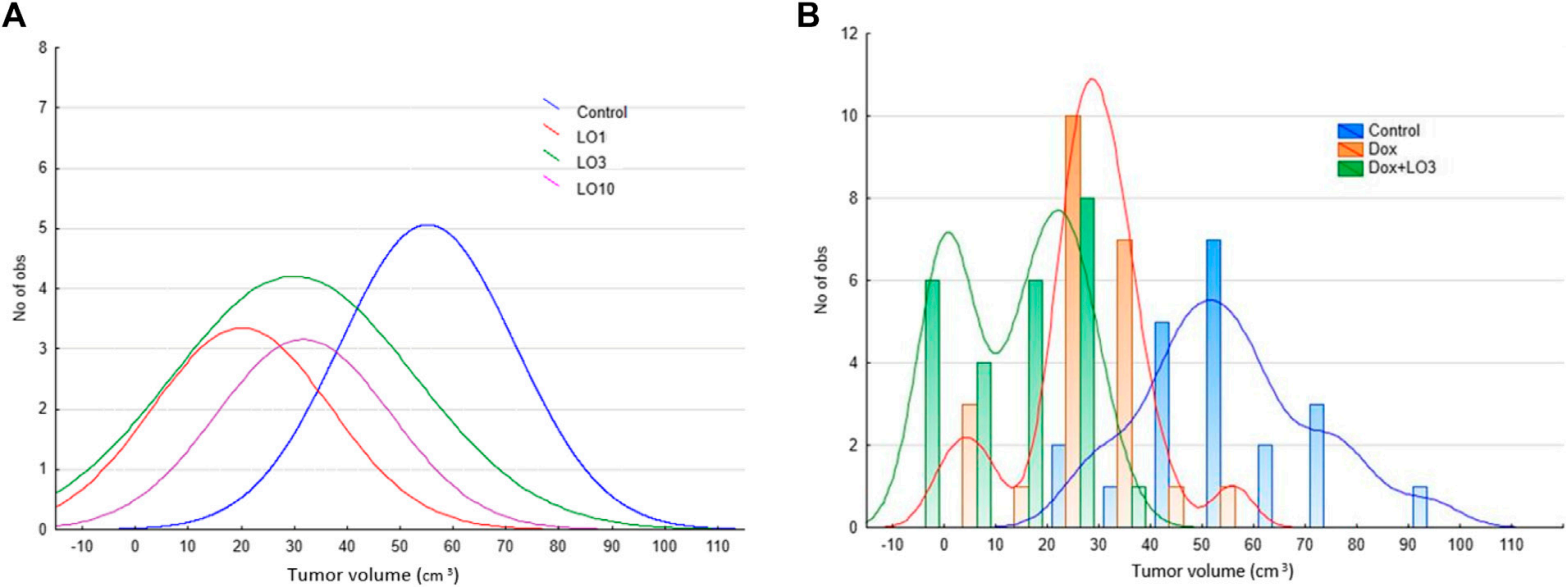
The frequency distribution of PLS tumor volumes 11 days after the start of treatment and the estimation of distribution density using Gaussian smoothing method **(A)** and Gaussian Kernel methods **(B)**.

Figures 3A,B shows the frequency distribution of tumor volume values at the end of treatment, and allow estimating the density of the distribution core. Thus, the distribution density for most probable *V*-value frequencies shifts from the range of 40–80 mm^3^ in the control group (95.2% of all data), to 0–55 mm^3^ in LO groups (Figure 3A). Interestingly, from Figure 3A it follows that CR can be reached by the end of monotherapy by LO (1, 3, and 10 ml/kg). As illustrated by Figure 3B, the distribution density for most probable frequencies in the case of Dox and Dox+LO 3 options falls on smaller values compared with the control group. It should be mentioned that among the groups indicated in Figure 3B, Dox+LO 3 was not characterized by a normal distribution (*p* = 0.002, Shapiro-Wilk test) but rather by a bimodal with the second mode appearing at zero tumor volume. So to compare the groups, the Gaussian Kernel density estimation method was used.

Based on data on the frequency of CR 60 days after the start of therapy, the values of the relative risk (RR) of tumor regression and the limits of its 95% CI were calculated for all the studied groups of animals receiving the studied treatment, relative to the control group that did not receive treatment. The data obtained are presented in Table 2.

**TABLE 2:** Quantitative assessment of the relationship between the treatment performed and the frequency of complete PLS regressions.

Parameter	Treatment groups						
	Dox	LO 1	LO 3	LO 10	Dox+LO 1	Dox+LO 3	Dox+LO 10
RR	5.139	4.353	8.222	4.353	4.353	9.962	4.353
−95% CI	0.631	0.423	1.050	0.423	0.423	1.303	0.423
+95% CI	41.859	44.775	64.389	44.775	44.775	76.183	44.775
RRR	4.139	3.353	7.222	3.353	3.353	8.962	3.353
RD	0.112	0.091	0.195	0.091	0.091	0.242	0.091
NNT	8.940	11.035	5.123	11.035	11.035	4.129	11.035
Chi-square	1.73	0.51	4.27	0.51	0.51	6.04	0.51
*p*-value	0.189	0.477	**0.039**	0.477	0.477	**0.014**	0.477
Phi-square	0.041	0.034	0.095	0.034	0.034	0.128	0.034

The values of *p* < 0.05 are highlighted in bold, which indicates statistically significant differences between the experimental and control groups. RR, Relative risk; RRR, Relative risk reduction; RD, Risk difference; NNT, Number needed to treat; Chi-square, Yates corrected Chi-square; -−95% CI, Lower limit (95% confidence interval); +95% CI, Upper limit (95% confidence interval).

The RR values for all experimental groups significantly exceed one and are maximum for the LO 3 and Dox+LO 3 groups, for which the probability of recovery increases by 8.22 and 9.96 times, respectively, compared with the control group (Table 2). The values of the Yates corrected Chi-square criterion indicates the reliability of differences in tumor regression in the experimental groups LO 3 and Dox+LO 3 in comparison with the control group (*p* < 0.05) and the presence of a relationship between the frequency of CR of PLS and the proposed treatment. Of note, the values of NNT, meaning the number of animals, which accounts for 1 case of CR, in the treatment of Dox (NNT = 8.94) by 1.7 and 2.2 times greater than in the treatment using LO 3 and its combination with Dox, respectively. The values of the Phi-square coefficient used to assess the strength of the relationship between the two variables, in this case between the treatment, performed and the frequency of complete regressions of PLS tumors, although they have maximum values for the experimental groups LO 3 and Dox+LO 3 (0.095 and 0.128, respectively), indicate the presence of an insignificant positive relationship between the therapy performed and the frequency of CR.

Considering different criteria evaluating the therapeutic effect, the combined treatment with Dox and LO demonstrated the most pronounced antitumor activity in the model of PLS. The use of LO at the optimal dose (3 ml/kg) resulted in a significant enhancement of Dox cytostatic activity.

### The High Antitumor Activity of Linseed Oil and Fish Oil in Combination With Dox in the Treatment Lewis Lung Adenocarcinoma

In the second part of the experiment on laboratory animals (mice), the antitumor efficacy of Dox (5 mg/kg), LO (6 ml/kg), FO (6 ml/kg), and combinations of Dox with omega-3 substrates was studied in a comparative aspect. The content of omega-3 PUFA in the indicated LO and FO doses was (289.40 ± 15.48) mg/100 g (ALA) and (113.84 ± 4.32) mg/100 g (the sum of EPA and DHA), respectively. No statistically significant changes in animal body weight were observed for all types of the above effects.

**FIGURE 4:**
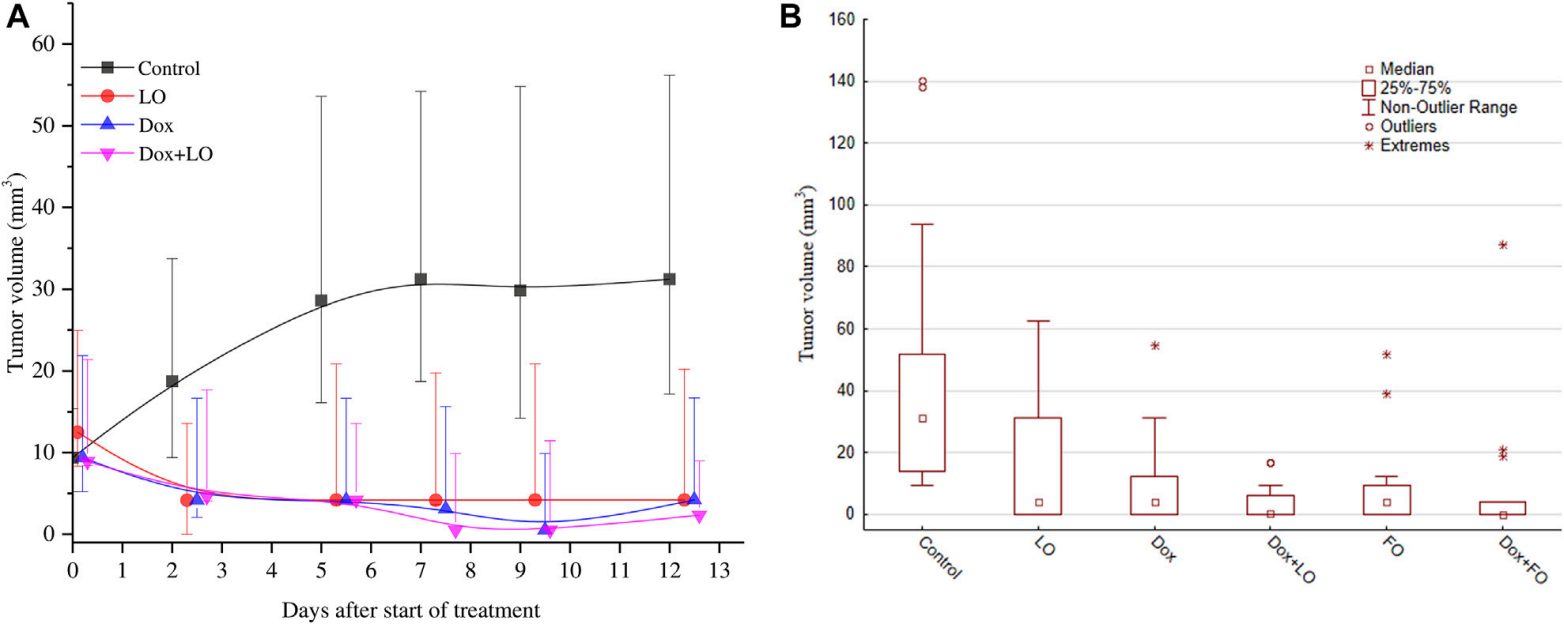
The dynamics changes of LLC tumor volume over the whole course of treatment **(A)**; the tumor volume 12 days after the start of treatment **(B)**. The results are obtained for all the studied groups in two independent experiments. The data is presented as the median and the values of the upper and lower quartile.

Figure 4 shows the dynamics changes of LLC volume for four groups of animals (Figure 4A) and the volumes 19 days after transplantation (12 days from the start of therapy) for the studied six groups of animals (Figure 4В). The data in the figures indicate a marked antitumor activity of LO, as well as Dox and its combination with LO during the entire observation period.

It can be noted from the calculations, that the experimental data obtained on LLC tumor volumes for the control group and all other groups treated do not have a normal distribution and are nonparametric, therefore median and nonparametric tests were used for multiple comparisons.

Table 3, Supplementary Table S3 provide descriptive statistics for tumor volume data at the end of treatment (12 days after the start of treatment) in all the studied groups, as well as the significance levels of pairwise comparison of tumor volume data according to the Mann-Whitney test.

**TABLE 3:** Descriptive statistics for the studied groups of animals according to values on the volume of LLC tumors after 12 days from the start of treatment.

Variable	Valid N	Median	Lower quartile	Upper quartile	Quartile range	Mean
Control	21	31.20	14.040	52.000	37.960	42.35
LO	17	4.200	0.000	31.2000	31.200	15.32
Dox	21	4.200	0.000	12.500	12.500	7.37
Dox+LO	20	2,330	0.000	6.640	6.640	6.02
FO	19	4.160	0.000	9.360	9.360	8.81
Dox+FO	20	0.00	0.000	4.160	4.160	7.41

N‒the number of animals in groups at the end of treatment. Died and euthanized animals are not included.

The obtained results indicate the presence of statistically significant differences in the values of the LLC tumors volume at the end of treatment when compared with intact control after all the above-mentioned treatment (*p* ≤ 0.001) (Supplementary Table S3). LO or FO daily administrated the average volume of tumors after 12 days of treatment decreased by 7.4–7.5 times compared to the control (*p* < 0.001). The volume of tumors after 12 days from the start of treatment using LO was statistically significantly different from the volume of tumors in combination treatment Dox+LO (*p* = 0.049), as well as Dox+FO (*p* = 0.015). At the same time, the group Dox did not differ statistically significantly in terms of tumor volumes from the groups with the combined use of Dox and LO, Dox, and FO.

**FIGURE 5:**
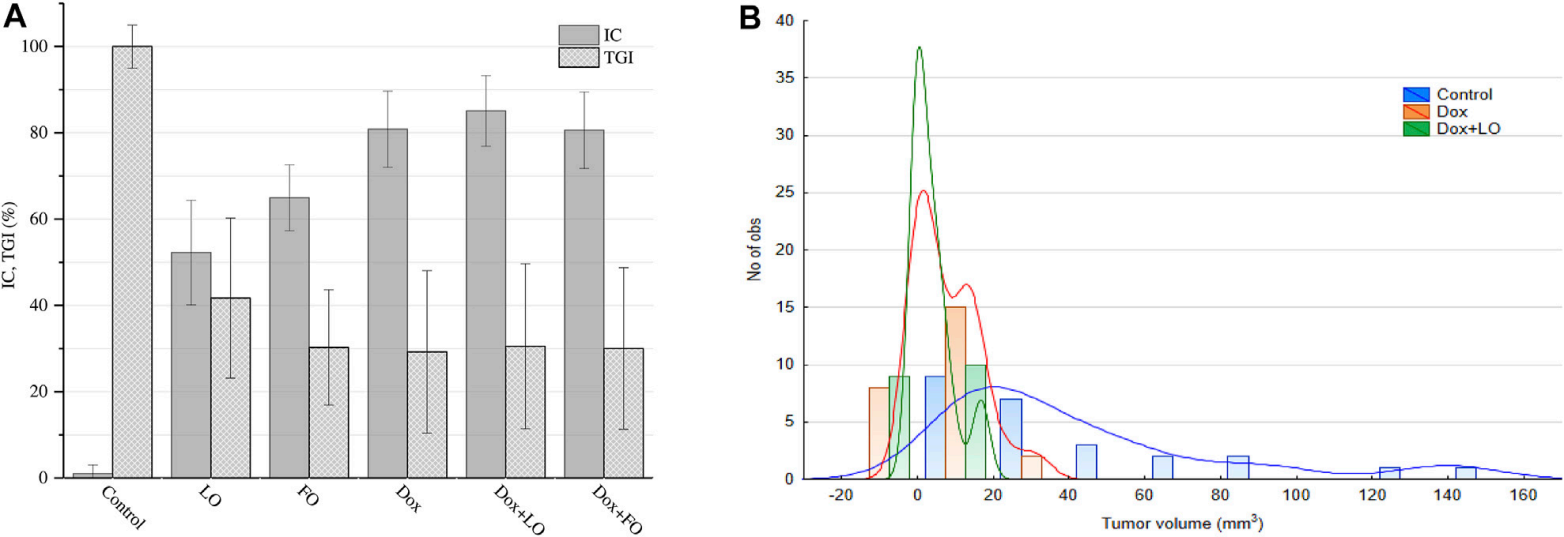
LLC tumor growth inhibition coefficient (IC) values 12 days after the start of treatment and tumor growth index (TGI) values corresponding to the whole treatment period **(A)**. The frequency distribution of tumor volumes 12 days after the start of treatment and the estimation of distribution density using Gaussian Kernel methods **(B)**. The values represent the mean % ± SE **(A)**. The data correspond to two independent experiments.

Figure 5A shows the IC and TGI values of 6-studied groups of animals. Analysis of IC values for groups of Dox, Dox+LO, and Do + FO made (80.82 ± 8.80)%, (85.07 ± 8.18)%, and (80.59 ± 8.84)%, respectively. According to the data obtained, the TGI values for the studied groups of animals treated with Dox, LO, FO, and their combinations differed statistically significantly from the control, but did not have statistically significant differences among themselves. When treated with Dox, FO, as well as a combination of Dox+LO, Dox+FO, TGI had minimal values that varied in the range of 29.2–30.5.

Figure 5B shows histograms of the frequency distribution of LLC volumes 12 days from the start of treatment and the **Gaussian** Kernel curves for the three studied groups of animals, allowing to estimate the density of the distribution core.

It can be seen from the data in Figure 5В that the core density of the distribution of the most probable frequencies for the Dox+LO group is maximal in the range 0–10 mm^3^, which accounts for 85% of all values. At the same time, for the Dox group, this interval (0–10 mm^3^) accounts for 69.2% of all values. The distribution density of the most probable frequencies for the control group is in the range from 10 to 100 m^3^ (92% of all values).

**FIGURE 6:**
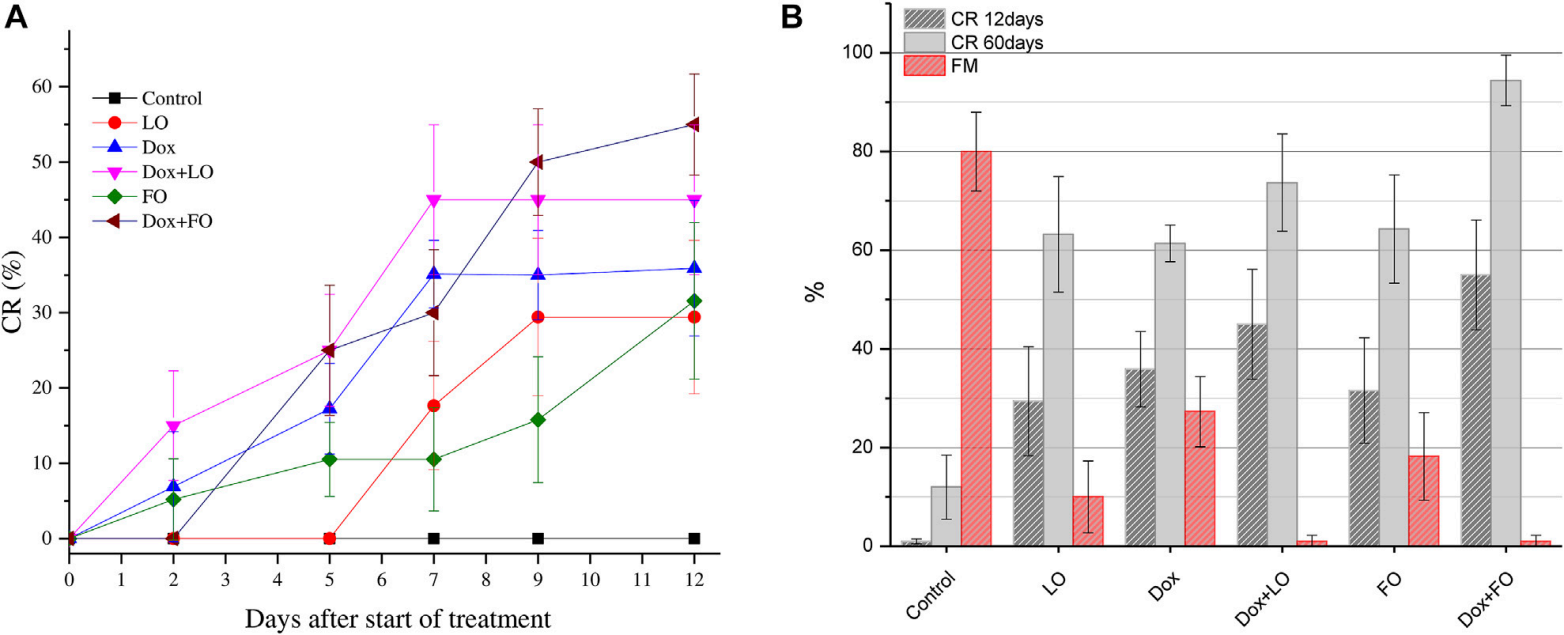
Change in the frequency of complete LLC regressions during treatment for all studied groups **(A)**; the CR value 12 and 60 days after the start of treatment and frequency of metastasis **(B)**.The values represent the mean ± 95% CI **(A)** and the mean % ± SE **(B)**. The data correspond to two independent experiments.

Figure 6 shows the dependences of the CR frequency of LLC on the time after the start of treatment (from the 7th to the 19th day after transplantation) (Figure 6A) and the CR values 12 and 60 days after the start of treatment and frequency of metastasis for six groups of mice (Figure 6B).

Figure 6A shows that, unlike the control group, in all experimental groups treated, the CR frequency growed with increasing duration of treatment and at the end of treatment was maximal in the groups treated with a combination of Dox+LO, Dox+FO [(45.00 ± 11.10)% and (55.05 ± 11.10)%, respectively]. Daily oral administration of LO and FO at a dose of 6 ml/kg, as well as only Dox at a dose of 5 mg/kg, provides a frequency of CR 61.37%–64.30% 60 days after the start of treatment. The use of Dox combined with LO and FO allows increasing the frequency of CR to (73.68 ± 9.82) and (94.4 ± 5.10)%, respectively. Statistically significant differences in the values of the frequency of CR of LLC tumors after 12 and 60 days from the start of treatment, when compared with intact control, occurred in all studied groups (*p* < 0.01, Student’s *t*-test). The frequency of CR 60 days after the start of treatment is significantly different for the LO, FO, and Dox groups when compared with the Dox+FO group (*p* ≤ 0.012). At the same time, the frequency of CR in the group receiving the combined treatment Dox+FO was 1.54 times higher than in the Dox group (*p* = 0.012, Student’s *t*-test).

Based on the data on the frequency of CR of LLC after 60 days from the start of treatment, the RR values of tumor regression and the limits of its 95% CI for all studied groups of animals receiving the studied treatment relative to the control group were calculated. The data obtained are presented in Table 4.

**TABLE 4:** Quantitative assessment of the relationship between the treatment performed and the frequency of complete LLC regressions.

Parameter	Treatment groups				
	LO	Dox	Dox+LO	FO	Dox+FO
RR	5.053	3.143	5.895	5.895	7.556
−95% CI	1.660	0.991	1.978	1.978	2.606
+95% CI	15.375	9.969	17.569	17.569	21.905
RRR	4.053	2.143	4.895	4.895	6.556
RD	0.507	0.268	0.612	0.612	0.819
NNT	1.974	3.733	1.634	1.634	1.220
Chi-square	9.85	10.74	14.15	14.15	24.5
*p*-value	**0.002**	**0.001**	**<0.001**	**<0.001**	**<0.001**
Phi-square	0.279	0.244	0.386	0.386	0.659

The values of *p* < 0.05 are highlighted in bold, which indicates statistically significant differences between the experimental and control groups. RR, Relative risk; RRR, Relative risk reduction; RD, Risk difference; NNT, Number needed to treat; Chi-square, Yates corrected Chi-square; −95% CI, Lower limit (95% confidence interval); +95% CI, Upper limit (95% confidence interval).

The RR values for all experimental groups significantly exceed 1 and are maximum for the FO group and the groups with combined treatment Dox+LO and Dox+FO, for which the probability of recovery increases by 5.89 and 7.56 times, respectively, compared with the control group (Table 4). The values of the Yates corrected Chi-square criterion confirm the reliability of differences in tumor regression in the experimental and control groups (*p* < 0.05) and the presence of a link between the frequency of CR LLC and the proposed treatment. At the same time, the values of NNT in the treatment of one Dox by 2.3 times greater than in the treatment of the Dox+LO and FO groups and by 3.1 times greater than in the Dox+FO group. The presence of a relationship between treatment in the test groups and the frequency of CR was also assessed by the Phi-square coefficient. Since the values of this coefficient for the Dox+FO group is 0.659, we can talk about the presence of a strong positive relationship between the therapy and the frequency of CR. The values of the Phi-square coefficient indicate the presence of a moderate (for Dox+LO and FO groups) and weak (LO and Dox groups) positive relationship between the treatment and the CR frequency.

Data on metastasis of experimental tumors (LLC) for one of the experimental series are presented in Supplementary Table S4; the values of the frequency of metastasis (FM) are also shown in Figure 6B. Of note, LO and FO showed high antimetastatic activity. The Dox+LO and Dox+FO groups demonstrated the highest antimetastatic activity [the average number of metastases in the groups was 0.00; and metastasis inhibition index (MII = 100.0%)].

Thus, the results of preclinical studies of LO and FO *in vivo* indicate their antitumor properties, as well as an increase in the antitumor activity of Dox when used in combination with omega-3 substrates.

## Discussion

It is known that the antitumor effect of Dox is mainly associated with its ability to penetrate the DNA of cancer cells, blocking their division (Siatis et al., 2010). In addition, having a quinoid fragment in its structure, Dox is a redox-active compound and participates in processes leading to an increase in the level of ROS, which can damage various biomolecules of cells (Doroshow, 1986). Assessing the contribution of free-radical processes to the formation of damage to cancer cells, it should be borne in mind that, unlike healthy ones, they multiply under conditions of sufficiently pronounced hypoxia. Therefore, during oncogenesis, oxidative processes are less likely to occur and the contribution to the destruction of biomolecules of reactions without oxygen increases.

In this regard, it is very important to have and analyze information about free-radical transformations of biologically important substances with different oxygen content. We have conducted studies of homolytic transformations of carbohydrates (Edimecheva et al., 2005), nucleic acid components (Petryaev and Shadyro, 1986), amino acids and peptides (Shadyro et al., 2003; Sladkova et al., 2012), glycerophospholipids (Yurkova et al., 2004; Yurkova et al., 2008), and sphingolipids (Lisovskaya et al., 2012) under various conditions. It was found that a characteristic feature of the free-radical transformations of the above compounds under hypoxia conditions is the fragmentation reaction of their radicals, which proceeds with the simultaneous rupture of two bonds located in the β-position to the radical center. It is shown that biomolecules containing hydroxyl and amino groups participate in this process. These are, first of all, cardiolipin—the main lipid of mitochondrial membranes, cerebrosides, sphingosine derivatives, mono- and polysaccharides. The implementation of fragmentation reactions leads not only to the destruction of the original molecules but also to the formation of substances that actively affect the processes of apoptosis and cell proliferation.

**SCHEME 1:**
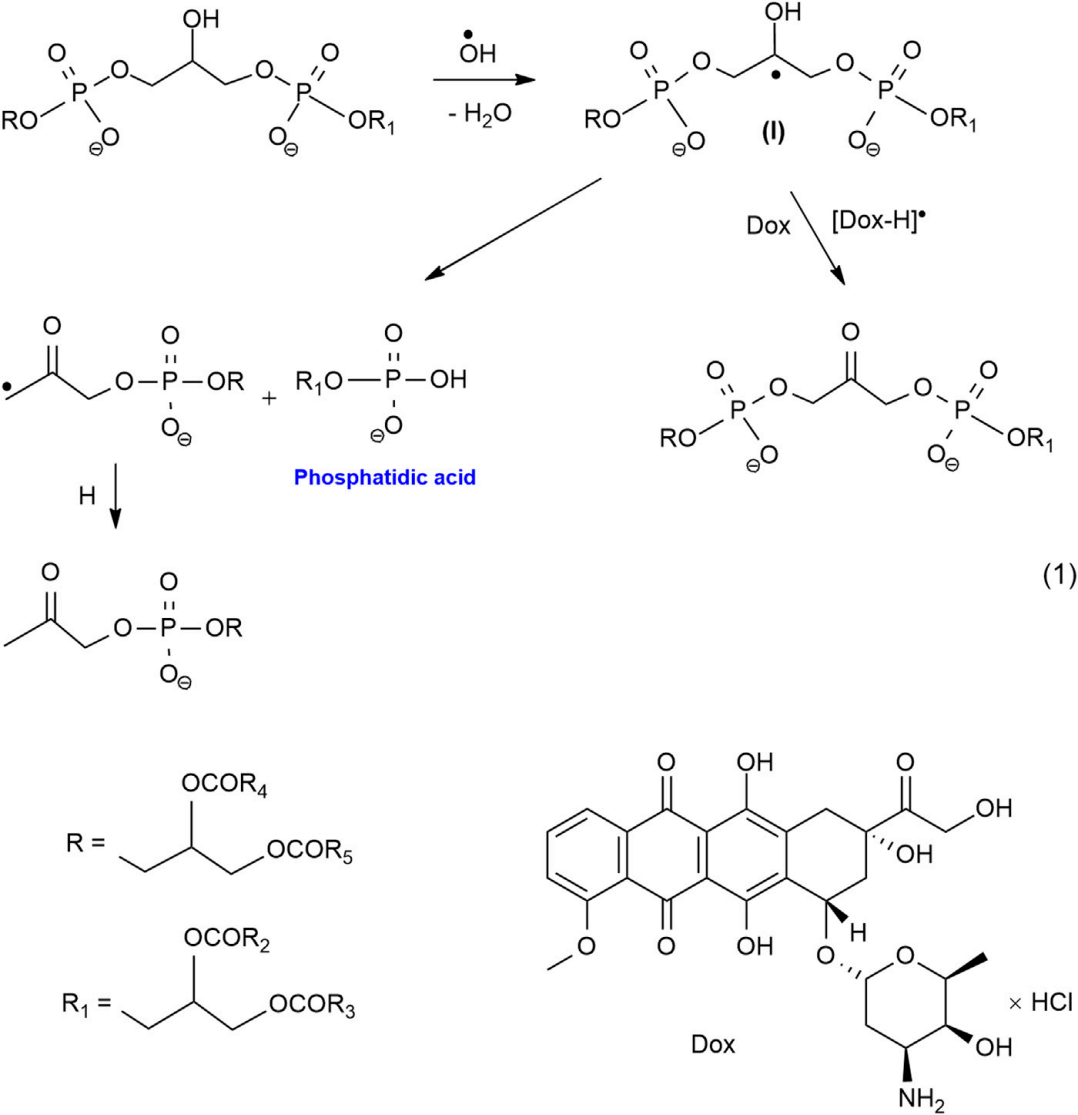


For the first time, we have established the fact of the formation of phosphatidic acid (PA) under the action of gamma radiation and redox systems on hydroxyl-containing glycerophospholipids, such as cardiolipin, phosphatidylglycerol, phosphatidylinositol (Shadyro et al., 2004; Yurkova et al., 2008). PA is known to play an important role in the regulation of cell proliferation and apoptosis in healthy cells (Wang et al., 2006). In recent years, it has been established that PA is a lipid messenger in the processes that contribute to the shutdown of apoptosis and increase the survival of cancer cells (Foster, 2009; Toschi et al., 2009). Consequently, the accumulation of PA will lead to a decrease in the effectiveness of chemo- and radiotherapy, therefore, a rational strategy for anticancer therapy should be aimed at regulating PA levels. In conditions of hypoxia, the best blocker of non-enzymatic pathways of PA formation, as shown in our works, are quinones (Dox, thymoquinone, and others), as well as substances containing a conjugated carbonyl group, such as curcumin, bioflavonoids, vitamins B, C and K (Shadyro et al., 2002; Shadyro et al., 2005; Brinkevich et al., 2012; Samovich et al., 2013). In the case of cardiolipin, the process of formation of PA due to the fragmentation of the radical (I) initial substrate and competitive oxidation of this radical by Dox, blocking the fragmentation of the radical (I) with the formation of PA can be represented by the following Scheme 1.

**SCHEME 2:**
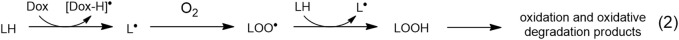


In the presence of rich omega-3, PUFA from LO Dox and other cytostatics-oxidants can activate the LPO processes of lipids LO (LH) in conditions of relative oxygen deficiency with the formation of cytotoxic products of oxidation and oxidative degradation, leading to irreversible damage to tumor cells. The ongoing processes can be represented by a simplified Scheme 2.

Thus, our proposed approach to the search for effective antitumor agents is based on the use of substances that actively regulate free-radical processes in hypoxia. Such regulation should ensure the blocking of processes that contribute to the survival of malignant cells and the intensification of reactions of the formation of products with cytostatic and cytotoxic properties, which will form an antitumor effect. This indicates the prospects of using antitumor drugs with oxidative properties, which include Dox (blockers of the biomolecule fragmentation reaction), in combination with PUFA of LO, FO (an easily oxidizing substrate) when creating new antitumor agents, and radiosensitizers for the treatment of oncological diseases.

The results of treatment of experimental tumors of Pliss lymphosarcoma and Lewis lung adenocarcinoma obtained in this work confirm the effectiveness of combination therapy using the quinoid cytostatic Dox and LO. LO administered daily orally for 11–12 days at doses of 1–10 ml/kg of animal body weight, inhibits tumor growth and increases the frequency of their complete regressions compared to the control, and also exhibits high antimetastatic activity. In combination therapy, LO enhances the antitumor effect of Dox, statistically significantly reducing the size of tumors and their metastatic activity, increasing the frequency of CR compared to groups treated with Dox alone.

The results of this work will be useful in conducting further studies to establish the possibility of using lower doses of Dox in the treatment of cancer when using their combination with LO, while providing high antitumor activity.

## Conclusion

The antitumor activity of LO in a wide dose range was studied in animal models with PLS and LLC tumors by mono and combination therapy with Dox.

It was found that the oral administration of LO (as a source of omega-3 PUFA) to rats daily at doses of 1, 3 and 10 ml/kg of body weight from 7 days after subcutaneous transplantation of PLS has a stable statistically significant effect on the dynamics of tumor growth, reducing the intensity of tumor growth and increasing the number of CR compared with the control. LO decreased the volume of PLS tumors of rats by 1.7–2.8 times compared to the control (*p* < 0.001, Tukey’s test), the CR frequency varied in the range of 14.28%–15.38% 11 days after the start of treatment. The frequency of CR was maximum after 60 days (22.20 ± 15.18)% for LO at a dose of 3 ml/kg.

LO at a dose 3 ml/kg potentiates the antitumor effect of Dox in the PLS model, reducing the volume of tumors after 11 days from the start of therapy by 2.0 times (*p* = 0.013, Tukey’s test), the value of the TGI by 1.6 times (*p* < 0.03, Student’s *t*-test) and increasing the frequency of CR after 60 days from the start of therapy by 3.5 times (from 7.7 % to 26.9%) (*p* = 0.019, Student’s *t*-test) compared with the use of Dox alone.

Assessment of the risk of regression of PLS for the studied groups of animals compared with the control group does not allow us to draw an unambiguous conclusion based on the data obtained that there is a connection between the treatment and the frequency of CR of PLS.

Daily administration of LO and FO to mice at a dose of 6 ml/kg of body weight from 7 days after subcutaneous transplantation of LLC, has a statistically significant effect on the growth dynamics of the tumor, reducing the intensity of tumor growth and increasing the number of their complete regressions. In groups of mice treated daily with LO or FO, the average volume of tumors at the end of treatment decreased by 7.4–7.5 times compared to the control (*p* < 0.001), the frequency of CR 60 days after the start of treatment was 61.37%–64.30%. LO and FO also showed high antimetastatic activity: the metastasis inhibition index (MII) was 99.18% and 96.94%, respectively.

It was found that LO and FO potentiates the antitumor effect of Dox, by increasing the frequency of complete regressions and reducing the metastatic activity of LLC. The use of combinations of Dox with LO and FO in the treatment makes it possible to increase the CR frequency to (73.68 ± 9.82)% and (94.4 ± 5.10)%, respectively. With combined treatment, the average number of metastases in the groups was 0.00, the MII was 100.0%.

Assessment of the risk of regression of LLC tumors for the studied groups of animals compared with the control group showed a moderate or strong positive relationship between the treatment and the frequency of CR for groups treated with a combination of Dox with LO or FO, respectively.

The data obtained are the basis for the clinical study of LO as an adjuvant in chemotherapy regimens using Dox.

A new approach to the search for effective antitumor agents is proposed, based on the use of substances that actively regulate free-radical processes in hypoxia conditions, such as LO and FO PUFA (easily oxidizing substrate)—and quinone derivatives, as well as substances containing a conjugated carbonyl group in their composition, such as Dox (oxidant).

## Data Availability

The original contributions presented in the study are included in the article/Supplementary Material, further inquiries can be directed to the corresponding author.
