# Supplementary material for: In Vivo Antitumoral Effects of Linseed Oil and Its Combination With Doxorubicin
Source: Front Pharmacol. 2022 Jun 21;13:882197. doi: 10.3389/fphar.2022.882197 (PMC9254224; doi:10.3389/fphar.2022.882197)
Supplement: Supplementary file 1 [file DataSheet1.pdf]

Supplementary Material

Supplementary Table S1. Characteristics of LO and FO used in the experiments on animal models.

Composition	Used for the PLS model	Used for the LLC model	
	LO	LO	FO
Fatty acids (g/100 g):			
C14:0 myristic	ND	ND	4.63 ± 0.20
C16:0 palmitic	5.64 ± 0.19	4.68 ± 0.19	12.20 ± 0.43
C16:1 (n-7) palmitoleic	ND	ND	9.27 ± 0.33
C18:0 stearic	4.72 ± 0.22	5.52 ± 0.18	1.60 ± 0.04
C18:1 (n-9) oleic	16.25 ± 0.72	20.76 ± 0.63	23.71 ± 0.74
C18:2 (n-6) linoleic	16.47 ± 0.73	17.03 ± 0.70	ND
C18:3 (n-3) linolenic	55.92 ± 2.90	51.82 ± 2.77	2.21 ± 0.08
C18:4 (n-3) stearidonic			0.10 ± 0.01
C20:0 arachidic			0.21 ± 0.01
C20:1 (n-9) eicosenoic			13.21 ± 0.56
C20:5 (n-3) eicosapentaenoic			7.80 ± 0.29
C22:1 (n-11) cetoleic			10.43 ± 0.42
C22:6 (n-3) docosahexaenoic			9.86 ± 0.38
Other	0.80 ± 0.05	0.45 ± 0.01	2.67 ± 0.12
n-3 PUFAs, total	55.92 ± 2.90	51.82 ± 2.77	19.97 ± 0.76
PUFAs, total	72.39 ± 4.88	68.85 ± 4.55	19.97 ± 0.76
Tocopherols, total (mg/100 g)	57.11 ± 2.71	50.13 ± 2.40	–
Vitamin A (IU/g)	–	–	8.0
Carotenoids, total (mg/100 g)	3.04 ± 0.16	2.67 ± 0.13	–
Coenzymes Q10 and Q9, total (mg/100 g)	6.25 ± 0.51	4.53 ± 0.37	–
Vitamin D3 (IU/g)	–	–	< 10
Phytosterols, total (mg/100 g)	574.74 ± 20.22	524.40 ± 19.58	–
PV (meq O_2/kg)	0.75 ± 0.04	1.22 ± 0.07	0.82 ± 0.04
AV (mg KOH/g)	0.60 ± 0.03	0.90 ± 0.04	0.12 ± 0.01
p-AnV	0.47 ± 0.02	0.45 ± 0.02	NA
IV (g I_2/100 g)	192.5 ± 9.2	184.7 ± 9.8	160 ± 4.2

Abbreviations: ND = not detected, defined as ≤ 0.05%; NA = not applicable or not available; IU = international unit; PV = peroxide value; AV = acid value; *p*-AnV = *para*-anisidine value; IV = iodine value; meq = milligram-equivalent. The results are expressed as the mean ± SD (n = 3).

Supplementary Table S2. Matrix of intergroup comparisons by the Tukey test of PLS tumor volume samples 11 days from the start of treatment. The values of $p \leq 0.05$ are highlighted in bold, which indicate significant intergroup differences.

**Treatment groups**	**Tukey HSD test; Approximate Probabilities for Post Hoc Tests**							
	**Control**	**Dox**	**LO 1**	**LO 3**	**LO 10**	**Dox+ LO 1**	**Dox+ LO 3**	**Dox+ LO 10**
Control		**< 0,001**	**< 0,001**	**< 0,001**	**< 0,001**	**< 0,001**	**< 0,001**	**< 0,001**
Dox	**< 0,001**		0,753	0,993	0,993	0,999	**0,013**	0,789
LO 1	**< 0,001**	0,753		0,393	0,393	0,549	0,876	1,000
LO 3	**< 0,001**	0,993	0,393		1,000	1,000	**0,005**	0,425
LO 10	**< 0,001**	0,993	0,393	1,000		1,000	**0,005**	0,425
Dox+LO 1	**< 0,001**	1,000	0,549	1,000	1,000		**0,011**	0,587
Dox+LO 3	**< 0,001**	**0,013**	0,876	**0,005**	**0,005**	**0,011**		0,815
Dox+LO 10	**< 0,001**	0,789	1,000	0,425	0,425	0,587	0,815	

Supplementary Table S3. Matrix of intergroup comparisons by the Mann-Whitney test of LLC tumor volume 12 days from the start of treatment. The values of $p \leq 0.05$ are highlighted in bold, which indicate significant intergroup differences.

**Variable**	**Mann-Whitney U-test (rank sum test), p-value**					
	**Control**	**LO**	**Dox**	**Dox+LO**	**FO**	**Dox+FO**
Control		**0.001**	**< 0.001**	**< 0.001**	**< 0.001**	**< 0.001**
LO	**0.001**		0.383	**0.049**	0.290	**0.015**
Dox	**< 0.001**	0.383		0.366	0.895	0.117
Dox+LO	**< 0.001**	**0.049**	0.383		0.399	0.613
FO	**< 0.001**	0.290	0.895	0.399		0.180
Dox+FO	**< 0.001**	**0.015**	0.117	0.613	0.180	

Supplementary Table S4. Data on metastasis in laboratory animals with LLC.

**Variable**	**The total number of metastases in the group, units**	**Frequency of metastasis (FM), %**	**Average number of metastases, units**	**The degree of lung metastasis**	**Metastasis inhibition index (MII), %**
Control	61	8 out of 10 = 80.0 %	6.10	1; 1; 1; 1; 1; 1; 1; 2	
LO	4	1 out of 10 = 10.0 %	0.40	1	99.18
FO	9	2 out of 11 = 18.2 %	0.82	1; 1	96.94
Dox	10	3 out of 11 = 27.3 %	0.91	1; 1; 1	94.91
Dox+LO	0	0 out of 9 = 0.0 %	0.00	ns	100.00
Dox+FO	0	0 out of 11 = 0.0 %	0.00	ns	100.00
